# Long chain n-3 polyunsaturated fatty acids and vascular function in patients with chronic kidney disease and healthy subjects: a cross-sectional and comparative study

**DOI:** 10.1186/s12882-016-0393-5

**Published:** 2016-11-21

**Authors:** Morten Borg, My Svensson, Johan V. Povlsen, Erik B. Schmidt, Christian Aalkjær, Jeppe H. Christensen, Per Ivarsen

**Affiliations:** 1 Department of Biomedicine, Aarhus University, Aarhus, Denmark; 2 Department of Respiratory Medicine and Allergology, Aarhus University Hospital, Nørrebrogade 44, build. 2A, 8000 Aarhus C, Denmark; 3 Department of Internal Medicine, Horsens Regional Hospital, Horsens, Denmark; 4 Department of Nephrology, Akershus University Hospital, Lørenskog, Norway; 5 Department of Nephrology, Aarhus University Hospital, Aarhus, Denmark; 6 Department of Clinical Medicine, Faculty of Health, Aarhus University, Aarhus, Denmark; 7 Department of Cardiology, Aalborg University Hospital, Aalborg, Denmark; 8 Department of Nephrology, Aalborg University Hospital, Aalborg, Denmark

**Keywords:** Polyunsaturated fatty acids, Hemodynamic, Resistance artery, Chronic kidney disease

## Abstract

**Background:**

Patients with chronic kidney disease have a markedly increased cardiovascular mortality compared with the general population. Long chain *n*-3 polyunsaturated fatty acids have been suggested to possess cardioprotective properties. This cross-sectional and comparative study evaluated correlations between hemodynamic measurements, resistance artery function and fish consumption to the content of long chain *n*-3 polyunsaturated fatty acids in adipose tissue, a long-term marker of seafood intake.

**Methods:**

Seventeen patients with chronic kidney disease stage 5 + 5d and 27 healthy kidney donors were evaluated with hemodynamic measurements before surgery; from these subjects, 11 patients and 11 healthy subjects had vasodilator properties of subcutaneous resistance arteries examined. The measurements were correlated to adipose tissue *n*-3 polyunsaturated fatty acids. Information on fish intake was obtained from a dietary questionnaire and compared with adipose tissue *n*-3 polyunsaturated fatty acids.

**Results:**

Fish intake and the content of *n*-3 polyunsaturated fatty acids in adipose tissue did not differ between patients and controls. *n*-3 polyunsaturated fatty acid levels in adipose tissue were positively correlated to systemic vascular resistance index; (*r* = 0.44; *p* = 0.07 and *r* = 0.62; *p* < 0.05, chronic kidney disease and healthy subjects respectively) and negatively correlated to cardiac output index (*r* = −0.69; *p* < 0.01 and *r* = −0.50; *p* < 0.05, chronic kidney disease and healthy subjects respectively). No correlation was observed between *n*-3 polyunsaturated fatty acid levels in adipose tissue and vasodilator properties in resistance arteries. *n*-3 PUFA content in adipose tissue increased with increasing self-reported fish intake.

**Conclusions:**

The correlations found, suggest a role for *n*-3 polyunsaturated fatty acids in hemodynamic properties. However, this is apparently not due to changes in intrinsic properties of the resistance arteries as no correlation was found to *n*-3 polyunsaturated fatty acids.

## Background

Patients with chronic kidney disease (CKD) have a high risk of cardiovascular disease (CVD) compared with the general population and with declining renal function, the risk of CVD increases [1]. In the general population, cardiovascular mortality has been reduced substantially during the past years, but in patients with CKD the high mortality from CVD remains unchanged [2]. The mechanisms responsible for the accelerated atherosclerosis in CKD are somewhat distinct from patients with classic CVD with intima lesions. They include inflammatory processes leading to vascular and myocardial fibrosis and vascular media calcifications [3, 4]. Although renal transplantation halts the progression of CVD, it still remains the leading cause of death after renal transplantation [5]. Large artery stiffness, as estimated by either pulse wave velocity (PWV) or augmentation index, has proved to be an independent predictor of CVD in patients with CKD [6].

Long chain *n*-3 polyunsaturated fatty acids (PUFA) might have cardioprotective effects [7, 8], and several studies have suggested a beneficial effect of long chain *n*-3 PUFA on CVD, although data are not entirely consistent [9–11]. The most important *n*-3 PUFA, eicosapentaenoic acid 20:5 (EPA) and docosahexaenoic acid 22:6 (DHA), are primarily found in fatty fish and are also available in fish oil supplements.

Several trials in non-CKD populations have addressed whether *n*-3 PUFA supplementation has an effect on arterial stiffness [12–14], and a meta-analysis concluded that *n*-3 PUFA supplementation significantly reduces arterial stiffness when determined as PWV or systemic arterial compliance [15]. Studies on the immediate effect of a large intake of *n*-3 PUFA have also proved an attenuating effect on arterial stiffness [16]. Contradicting results have emerged from trials testing the effect of *n*-3 PUFA supplementation on the vascular endothelial function, evaluated as flow-mediated dilatation of the brachial artery. Thus, *n*-3 PUFA improved the endothelial function in patients with metabolic syndrome, whereas such an effect could not be documented in healthy adults [17, 18]. The resistance arteries of the body are of critical importance for blood pressure regulation, organ perfusion and peripheral vascular resistance [19] and are presumed to be of major importance in the development of CVD. Previous investigations have not shown difference in morphology or vasoconstrictor response when comparing CKD and control resistance arteries [20], whereas studies on endothelial function have shown contradicting results [21–24].

The aim of this explorative cross-sectional and comparative study of patients with CKD stage 5 + 5d and healthy kidney donors was to correlate *n*-3 PUFA concentration in adipose tissue with arterial stiffness, cardiac output, systemic vascular resistance and endothelium-mediated vasodilatation in resistance arteries. Furthermore self-reported fish intake was compared to *n*-3 PUFA concentration in adipose tissue.

## Methods

### Study population

A total of 18 patients with chronic kidney disease stage 5 + 5d (CKD) and 27 healthy kidney donors were included in the study. One patient was excluded from statistical analysis because of missing values for *n*-3 PUFA in adipose tissue. Inclusion criteria were age above 18 years and scheduled for either living related-donor renal transplant (14 patients) or insertion of peritoneal dialysis catheter (3 patients) at the Department of Nephrology, Aarhus University Hospital, Denmark, during 1st of November 2010 until the 31st of October 2011. Inclusion ran consecutively and patients were approached when an operation date was scheduled. Exclusion criteria were severe congestive heart failure, persisting cardiac arrhythmias, reduced pulmonary function, leg amputation, severe psychiatric disease and acute infection. Three patients had diabetes mellitus, and 3 had previously documented CVD. Ahead of inclusion nine patients were not on dialysis, while 8 were treated with peritoneal dialysis. Median dialysis vintage was 294 days (range: 100–1154 days). All but one of the patients were treated with one or more antihypertensive drug: ACE-inhibitor (6 patients), angiotensin II receptor antagonist (7 patients), calcium channel antagonist (13 patients), beta-blocker (8 patients), furosemide (15 patients).

Patients prepared for living donor transplantation received immunosuppressive therapy 2 days in advance: Prednisolone 20 mg/d, Tacrolimus 0.2 mg/kg/d and Mycophenolat mofetil acid 1.5 g/d.

Twenty-seven healthy kidney donors who did not receive any medication served as controls. Control subjects were related to the patients (sibling, husband, wife, mother, father or friend).

The day before surgery, the participants’ hemodynamic data were measured along with height, weight and blood pressure. The participants filled out a questionnaire regarding fish intake, and fasting blood samples were drawn on the day of surgery. During surgery, a 2 × 3 cm biopsy from the abdominal wall containing skin, adipose tissue and subcutaneous resistance arteries was removed. Adipose tissue was submerged in liquid nitrogen and stored in a −80 °C freezer prior to analysis of fatty acid composition. Resistance arteries from the biopsy were isolated and mounted for isometric force measurements.

### Hemodynamic study

Seventeen patients with CKD and 27 healthy subjects were part of the hemodynamic study. Blood pressure, pulse rate and cardiac output (CO) were measured with a portable non-invasive device consisting of a three-way respiratory valve with a mouthpiece and a rebreathing bag connected to an infrared photoacuostic gas analyzer, a pulse oxymeter and a device for automatic blood pressure measurement (Innocor®, Innovision, Odense, Denmark). CO was measured by rebreathing a gas mixture of sulfahexafluoride (SF_6_, 0.1%) and nitrous oxide (N_2_0 0.5%) in an O_2_/N_2_ mixture. Rebreathing was done in 15 s with a gas volume of 1.8 l and a respiratory breathing rate of 14–18 min^−1^. Gas was sampled continuously from the mouthpiece and analyzed on-line by the IR gas analyzer. A constant ventilation rate and volume were ensured by synchrony between the graphical tachymeter on the computer screen and the study subject, who was instructed to empty the bag with each breath. The rebreathing software calculated pulmonary blood flow from the rate of uptake of N_2_O into the blood (slope of regression line through logarithmically transformation of expiratory N_2_O concentration plotted against time). The first two or three breaths were excluded from analysis if total lung volume changes measured by SF_6_ indicated incomplete gas mixing. After correction for system volume changes using SF_6_ concentration, the first two or three breath were excluded from the analysis due to initial incomplete gas mixing. For the majority of patients without pulmonary arterial-venous shunt (S_aO2_ ≥ 98%) the measured pulmonary blood flow value was considered equal to CO, whereas for patients with pulmonary shunt, the shunt was calculated and added to CO. The shunt fraction was calculated using the oxygen concentration. The calculations were performed under the assumption that gasses were mixed completely, that equilibration of gasses between alveoli and blood was rapid and that lung blood flow was constant.

Systolic blood pressure and diastolic blood pressure were measured using an automatic device connected to the Innocor®. Systemic vascular resistance index (SVRi) was calculated as: (Mean arterial blood pressure—central venous pressure)/Cardiac output and indexed to body surface. The measurement was performed twice, and the mean value was used for data analysis.

Pulse wave velocity (PWV) and augmentation index (AI) were measured in the supine position after 10 min of rest. Carotid–femoral PWV was measured with SphygmoCor® (AtCor Medical, TX, US), using the integral software.

Augmentation pressure was calculated as the difference between the second and first systolic peaks, and AI was calculated as the augmentation pressure expressed as percentage of pulse pressure. AI was measured for aorta. All of the measurements were made in duplicate by one trained study nurse, and the mean values were used in the subsequent analysis.

### Microvascular study

In the microvascular study, 11 patients with CKD and 11 healthy subjects were examined; the included patients where those where dissection were successful and viable vessels found. Skin and subcutaneous tissue samples were taken during surgery and immediately placed in a 5 °C physiological salt solution (PSS). In the biopsies, 2 mm long resistance artery segments were dissected for measurement of endothelium-dependent vasodilatation. The artery segments were mounted on two stainless steel wires (40 μm diameter) in organ baths of a 4-channel wire myograph (model 610M, Danish Myo Technology, Aarhus, Denmark) or in a 2-channel wire myograph (model 410A, Danish Myo Technology) for isometric force measurements. The organ baths contained PSS at 37 °C, continuously bubbled with 5% CO_2_ in air to keep pH at 7.4. After mounting, the arteries equilibrated for 20 min before the elastic properties were characterized by stepwise increasing the artery circumference as previously described [25]. Experiments were performed at 90% of L100; where L100 is defined as the circumference of the relaxed artery when exposed to a transmural pressure of 100 mmHg. The artery viability was tested twice with 10 μM noradrenaline (NA) before beginning the protocol. Upon preconstriction with 3 μM NA, a concentration-response curve was performed with increasing concentrations of the endothelium-dependent vasodilator acetylcholine (ACh). After washout and 20 min of rest, the experiment was repeated in the presence of the cyclooxygenase(COX)-inhibitor indomethacin (3μM), and after another 20 min of rest, the protocol was repeated in the presence of indomethacin and the nitric oxide-synthase inhibitor NG-nitro-L-arginine methyl ester (L-NAME) (100 μM). Hence “total” endothelial function, non-COX-dependent and non-COX/nitric oxide-dependent endothelial function was tested, while nitric oxide-dependent endothelial function was calculated.

The composition of PSS: NaCl 119, KCl 4, KH_2_PO_4_ 1.18, MgSO_4_ 1.17, NaHCO_3_ 25, CaCl_2_ 1.6, EDTA 0.026, glucose 5.5 (mM). Indomethacin was dissolved in ethanol, all other chemicals in distilled water. Chemicals were acquired from Sigma-Aldrich (St. Louis, MO, US).

### Evaluation of fish intake

Self-reported fish intake was obtained using a previously validated dietary questionnaire [26]. A score was given according to fish consumption at lunch and dinner as follows: 1 = never eating fish; 2 = eating fish once a month; 3 = eating fish twice a month; 4 = eating fish once a week; 5 = eating fish 2 to 3 times a week; 6 = eating fish every day. Fish scores were divided into three groups as low (2–5), moderate (6–8) and high (9–12) fish intake for further analysis.

### Analysis of fatty acid composition of adipose tissue

Adipose tissue samples were obtained from the abdominal region and stored at −80 °C. Fatty acids were extracted in CHCl_3_/MeOH [27], dissolved in hexane and transesterified using 0.5 M of sodium methoxide and acetic acid [28]. Fatty acid analysis was performed by gas chromatography using a Chrompack CP-9002 gas chromatograph (Chrompack Int, Middelburg, the Netherlands) and expressed as percentage of total fatty acids.

### Biochemistry

Estimated glomerular filtration rate (eGFR) was calculated from the 4-point MDRD formula [29]. Asymmetric dimethyl arginine (ADMA) was measured using ELISA (DLD Diagnostika GmbH, Germany). All other biochemistry was analysed at the Department of Clinical Biochemistry, Aarhus University Hospital.

### Statistics

Data in tables are presented as mean ± standard deviation. Baseline characteristics were compared using Student’s *t*-test or rank-sum when appropriated; gender was compared using Fisher’s exact test. Normality was visual check of quintiles of the individual variables against quintiles of normal distribution and standardized normal probability plot and using Sharpiro-Wilks test. All 3 tests should suggest normality for it to be accepted. Only *n*-3 PUFA was not normally distributed and had to be logarithmically transformed and normality was confirmed by Sharpiro-Wilks test. Log *n*-3 PUFA concentrations in adipose tissue had equal median and variance for CKD patients and healthy subjects, and were pooled and divided into three fish intake groups, which were compared using Kruskal-Wallis test. Correlation tests were conducted using linear regression. Comparison of the regression coefficient for healthy subjects and CKD patients was done as described by the Educational Department of UCLA [30]. All statistical analyses were performed using STATA v. 13 SE (STATA Corp., TX, US).

## Results

### Study population

Baseline statistics are seen in Table 1. Fish score was similar in CKD patients and healthy subjects (CKD 7.5 ± 2.0, *n* = 17 vs. healthy subjects 7.1 ± 2.4, *n* = 27). Content of long chain *n*-3 PUFA in adipose tissue were comparable between CKD patients and healthy subjects (CKD 0.25 ± 0.18%, *n* = 17 vs. healthy controls 0.29 ± 0.21%, *n* = 23).

**Table 1:** Baseline characteristics given as mean ± standard deviation

Variable	CKD	Healthy
N	17	27
Age (years)	45(20–74)	52(25–70)
Sex (male/female)	9/8	9/18
Systolic blood pressure (mmHg)	128 ± 12	121 ± 17^a^
Diastolic blood pressure (mmHg)	77 ± 9	74 ± 9^a^
BMI (kg/m^2^)	25.8 ± 4.3	24.9 ± 3.2^a^
eGFR (ml/min/1.73 m^2^)	6 ± 2*	84 ± 14
Hemoglobin (mmol/l)	7.0 ± 0.7*	8.4 ± 0.9
ADMA (μmol/l)	0.75 ± 0.09	0.70 ± 0.09
Calcium ion (mmol/l)	1.18 ± 0.11	1.20 ± 0.06
Phosphate (mmol/l)	1.76 ± 0.37*	1.11 ± 0.13
Parathyroid hormone (pmol/l)	22.4 ± 14.6	n.a.

*BMI* Body Mass Index, *eGFR* Estimated glomerular filtration rate—eGFR in CKD is from non-dialysis patients, *ADMA* Asymmetric dimethyl arginine

*p < 0.05

^a^ *n* = 26

PWV was higher in CKD patients (CKD 9.53 ± 4.08 m/s, *n* = 15 vs. 7.24 ± 1.46 m/s, *n* = 25; *p* < 0.05), augmentation index was lower in CKD patients (CKD 70.5 ± 22.6%, *n* = 15 vs. healthy subjects 86.3 ± 17.5%, *n* = 25; *p* < 0.01) while cardiac output index and SVRi did not differ significantly between CKD patients and healthy subjects (Cardiac output index —CKD 3.25 ± 0.94 L/min/m^2^, *n* = 16 vs. healthy subjects 2.81 ± 0.66 L/min/m^2^, *n* = 26; SVRi —CKD 30.9 ± 9.85 mmHg/(L/min)/m^2^, *n* = 16 vs. 32.6 ± 6.9 mmHg/(L/min)/m^2^, *n* = 26). Baseline characteristics for the microvascular study group (11 vs. 11) did not differ from the whole group. No difference was seen between dialysis and non-dialysis patients (data not shown).

### Fish score groups and n-3 PUFA in adipose tissue

**Fig. 1:**
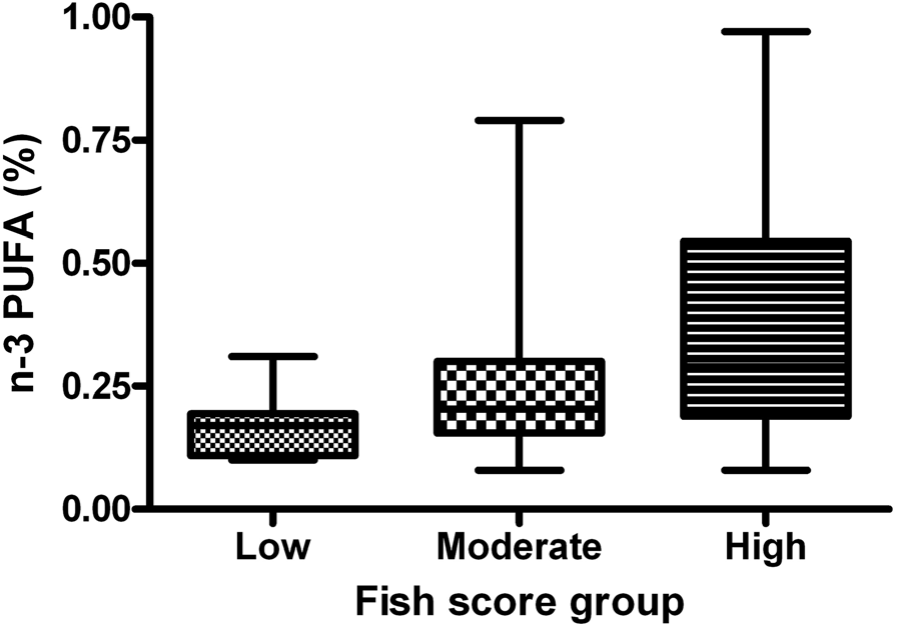
The participants’ fish score divided into three groups as low (2–5), moderate (6–8) and high (9–12). Each group’s content of *n*-3 PUFA in adipose tissue. The 3 groups significantly differ from each other (Box plot; (*p* < 0.01); Kruskal-Wallis test)

**Fig. 2:**
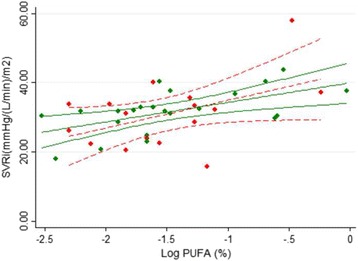
The association between *n*-3 PUFA in adipose tissue and systemic vascular resistance index. Chronic kidney disease patients (*r* = 0.44; *p* = 0.07) (*red symbols*/*red dash line*, *fitted line* and 95% confidence interval); healthy subjects (*r* = 0.62; *p* < 0.05) (*green symbols* and *solid line*). Regression formulas were y = 8.0*x + 43.0 and y = 6.3*x + 40.0 for CKD patients and healthy subjects, respectively

**Fig. 3:**
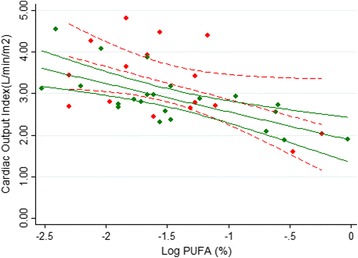
The association between *n*-3 PUFA in adipose tissue and cardiac output index. Chronic kidney disease patients (*r* = −0.69; *p* < 0.01) (*red symbols*/*red dash line*, fitted line and 95% confidence interval); healthy subjects (*r* = −0.50; *p* < 0.05) (*green symbols* and *green solid line*). Regression formulas were y = −0.79*x + 2.06 and y = −0.68*x + 1.87 for ESRD patients and control subjects, respectively

All subjects (*n* = 44) were divided into three groups according to their fish intake. As shown in Fig. 1, *n*-3 PUFA content in adipose tissue increased with increasing fish intake and groups were significantly different from each other (*p* < 0.01).

### Adipose *n*-3 PUFA and hemodynamic function

The content of long chain *n*-3 PUFA and SVRi was positively correlated in both CKD (*r* = 0.44; *p* = 0.07, *n* = 16) and control subjects (*r* = 0.62; *p* < 0.05, *n* = 26). No differences in hemodynamic function between CKD and controls were found (Fig. 2). Furthermore, *n*-3 PUFA were negatively associated with cardiac output index in both CKD and controls (*r* = −0.69; *p* < 0.01, *n* = 16 and *r* = −0.50; *p* < 0.05, *n* = 26; respectively) (Fig. 3).

### Adipose *n*-3 PUFA and microvascular function

**Fig. 4:**
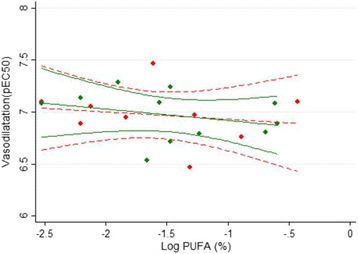
The association between *n*-3 PUFA in adipose tissue and acetylcholine-induced vasodilator response in resistance arteries. Chronic kidney disease patients *red symbols*/*red dash line*, *fitted line* and 95% coefficient interval); healthy subjects (*green symbols* and *green solid line*). Regression formulas were y = −0.07*x + 6.86 and y = −0.11*x + 6.81 for ESRD patients and control subjects, respectively

The “total” Ach-induced vasodilatation in the microvasculature was not associated with the content of *n*-3 PUFA in adipose tissue (Fig. 4). Furthermore, specific pathways such as non-COX-dependent, nitric oxide-dependent and non-COX/nitric oxide-dependent were not correlated to *n*-3 PUFA in adipose tissue (data not shown).

## Discussion

*n*-3 PUFA content in adipose tissue and self-reported fish intake were both similar in CKD patients and controls. In both CKD and healthy subjects, *n*-3 PUFA levels and SVRi were positively associated, whereas *n*-3 PUFA and cardiac output index were negatively associated. The content of long chain *n*-3 PUFA in adipose tissue was not associated with endothelial function in small resistance vessels.

### Fish intake and *n*-3 PUFA

*n*-3 PUFA content in adipose tissue (a long-term marker of *n*-3 intake [31]) and fish intake were similar in CKD patients and healthy controls. The majority of subjects were related to one of the CKD patients as a sibling, husband, wife, mother, father or friend, and hence a few of them shared household and therefore, the control group may tend to have the same fish intake. It should, however, be appreciated that the fish score gives a rather crude measure of consumption and particularly of intake of EPA and DHA, which is a limitation to the study. Previous studies have described that US hemodialysis (HD) patients have much lower fish intake than recommended [32] as well as lower levels of *n*-3 PUFA compared with controls [33]. In contrast, HD patients in South Korea [34] and peritoneal dialysis patients in Greece [35] had similar levels of *n*-3 PUFA compared with the background population. This elucidates the importance of regional differences in fish intake when comparing dialysis patients with the general population.

The duration of HD has also proved important for the general nutritional intake, since both total energy, protein and lipid consumption are negatively correlated with the duration of HD [36]. In the current study, both patients with CKD treated with dialysis and pre-dialysis patients were included. Participants were relatively young (median 45 years) with a median dialysis vintage of 294 days. The majority was undergoing renal transplant and hence in good condition with retained nutritional intake. This could help explain the similar fish intake between patients and controls seen in this study. Despite these considerations, the similarity in fish intake and *n*-3 PUFA levels between CKD patients and controls suggests that uptake, processing and storage of *n*-3 PUFA in CKD patients are comparable to healthy controls, as described earlier [37].

### Hemodynamic function

A positive correlation between *n*-3 PUFA in adipose tissue and the SVRi was found in both CKD patients and control subjects. An earlier study in healthy adults reported that resting SVR 5 hours after consuming a meal rich in DHA or EPA was the same as in the controls given a meal without DHA or EPA [38]. A subsequent exercise test resulted in a lower SVR in the DHA-consuming group compared with both the control and the EPA-consuming group. A negative correlation was found between the content of *n*-3 PUFA in adipose tissue and cardiac output index in both controls and CKD patients. The aforementioned study [38] of *n*-3 PUFA acute effects did not find differences in cardiac output at rest or exercise after DHA/EPA/placebo ingestion. Resting cardiac output is reduced in patients with CVD [39]. However, since a correlation was found in both CKD and healthy controls, the current results could also suggest that long-term fish intake enhances tissue oxygen uptake resulting in a lowering of the cardiac output, as suggested recently [40]. Moreover, in animal models it is well known that *n*-3 PUFA lower the basic metabolic rate [41], which could also result in a lowering of cardiac output. The diminished cardiac output could subsequently result in a baroreceptor-mediated augmentation in SVR. A major part of the vascular resistance resides in the microvasculature. However, since we found no correlation between *n*-3 PUFA and the microvascular endothelial-mediated vasodilator function, this apparently is not due to changes in intrinsic properties of the resistance arteries. Rather the change in resistance may stem from continual in-vivo activation from nerves, paracrine or endocrine processes.

The current study showed associations between *n-3* PUFA and cardiac index/SVRi. However, associations were not found with PWV or augmentation index. This contradicts an earlier study showing DHA to be inversely associated with PWV in non-diabetic CKD patients [42]. PWV is used as a marker of early-stage atherosclerosis [43], and the lack of coherence between results could possibly be explained by the young age of the CKD patients, the low dialysis vintage or our relatively small sample size.

It has been difficult to prove an effect of *n*-3 PUFA ingestion on mortality or CVD in CKD patients in clinical trials. However, an observational study [44] has suggested DHA in red blood cells to be an independent predictor of mortality in end stage renal disease patients on hemodialysis after 10 years of follow-up. Also, a clinical controlled trial found 2 years of *n*-3 PUFA treatment to be beneficial in secondary prevention of myocardial infarction in chronic HD patients, while no effect was seen on the primary endpoint, a composite of total cardiovascular events and death [45]. Longer prospective studies on large cohorts are needed to determine the effect of *n*-3 PUFA on hemodynamic function and mortality in CKD patients.

### Microvascular function

Although several studies have addressed the effect of *n*-3 PUFA on endothelial function in large arteries [17, 18], this study is the first to correlate the content of *n*-*3* PUFA in adipose tissue with microvascular function. The endothelial Ach-induced vasodilatation was not correlated to *n*-3 PUFA in adipose tissue. Furthermore, the individual Ach-induced vasodilator pathways were not associated with long chain *n*-3 PUFA levels (data not shown). These results are in line with animal studies performed on rat femoral resistance arteries [46] and suggest that the intrinsic properties of resistance arteries are not affected by *n*-3 PUFA.

The study design was cross-sectional and therefore cannot establish causal relationship between variables. Since all healthy kidney donors were related to an CKD kidney recipient, the two groups may tend to have the same fish intake. This could influence measurements of hemodynamic and microvascular properties and potentially mask differences which could have been found in non-related groups. Due to the rather low sample size, a risk of type II error exists. In this type of experiment confounding variables are also a hazard. Due to the size of the study, is has not been possible to adjust for confounders such as smoking, exercise or family history.

## Conclusions

In conclusion, this study found similar fish intake and adipose content of long chain *n*-3 PUFA in CKD patients and control subjects. This is in contrast with earlier findings, but might be explained by young age of subject and the short duration of dialysis. Long chain *n*-3 PUFA were negatively correlated to cardiac output index, which may imply that *n*-3 PUFA enhance tissue oxygen uptake and lower metabolic rate. This could further explain the observed positive correlation between *n*-3 PUFA and systemic vascular resistance index although not through intrinsic properties of resistance arteries, as they did not correlate to long chain *n*-3 PUFA in adipose tissue.

## Abbreviations

ACh: Acetyl choline
ADMA: Asymmetric dimethyl arginine
AI: Augmentation index
CKD: Chronic kidney disease
CO: Cardiac output
COX: Cyclooxygenase
CVD: Cardiovascular disease
DHA: Docosahexaenoic acid 22:6
eGFR: Estimated glomerular filtration rate
EPA: Eicosapentaenoic acid 20:5
HD: Hemodialysis
L-NAME: NG-nitro-L-arginine methyl ester
*n*-3 PUFA: *n*-3 Polyunsaturated fatty acids
NA: Noradrenaline
PSS: Physiological salt solution
PWV: Pulse wave velocity
SVRi: Systemic vascular resistance index
